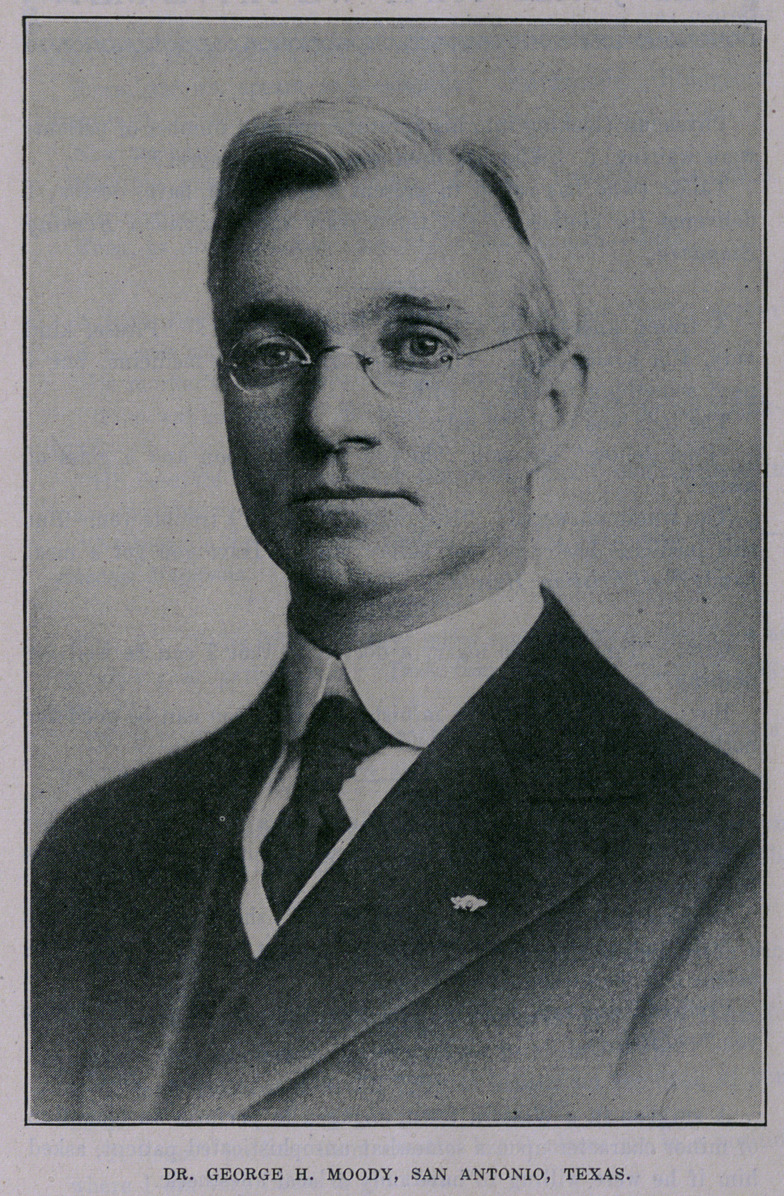# Dr. George H. Moody

**Published:** 1917-05

**Authors:** 


### Dr. George H. Moody.

Dr. George H. Moody, of San Antonio, Texas, died at his home on April 30, 1917.

There are many physicians in this State and in other States to whom the announcement of the death of Dr. George H. Moody will come as a distinct personal loss, for while Dr. Moody was a comparatively young man, his diversified activities had won for him a host of friends outside the medical profession. In the profession, no man was more generally loved than he, for his gentle bearing and his simple manner made him easily approachable and won for him the appellation, the "Friend of men."

It seems such a pity for such men to die; the world needs them so much.

Dr. Moody will be missed not only by his loved ones but by the host of friends to whom he was so dear.

He was born in Mexia, Texas, May 12, 1872. He was the son of James I. Moody and Eureka Moody. He graduated in medicine in the Medical Department of Tulane University, New Orleans, in 1896, and practiced medicine in Mexia, Texas, for a short time. He was assistant physician at the State Lunatic Asylum at Austin and afterward for four years was first assistant superintendent of the Southwestern Insane Asylum at San Antonio, from which position he resigned May 1, 1903, and continued his studies in nervous diseases and psychiatry in Europe. Upon returning he established "Dr. Moody's Sanitarium" for the treatment of nervous and mental diseases in November, 1903. He was a member of the American Medico-Psychological Association, an active member of the Bexar County and Fifth District Medical societies, and contributed a great many papers on psychiatry and neurology to these societies. He was President of the Bexar County Medical Society, the Fifth District Medical Society, and the Medical Association of the Southwest, and the State Medical Association. He was a member of the San Antonio Chamber of Commerce, a Knight Templar, a Shriner, an Elk, a Rotarian, etc. In 1907, he married Miss Bebe Denman, of San Antonio. Two sons were born of this union—George H. Moody, Jr., and Leroy Denman Moody.

The love and sympathy of many sorrowing friends goes out to Mrs. Moody and the children in this hour of their great sorrow.

## Figures and Tables

**Figure f1:**